# Synthesis and *in Vitro* and *in Vivo* Anticoagulant and Antiplatelet Activities of Amidino- and Non-Amidinobenzamides

**DOI:** 10.3390/molecules21050676

**Published:** 2016-05-21

**Authors:** Soo Hyun Lee, Wonhwa Lee, Jong-Sup Bae, Eunsook Ma

**Affiliations:** 1 College of Pharmacy, Catholic University of Daegu, Hayang-ro 13-13, Hayang-eup, Gyeongsan-si, Gyeongbuk 712-702, Korea; soohyun0320@cu.ac.kr; 2 College of Pharmacy, CMRI, Research Institute of Pharmaceutical Sciences, Kyungpook National University, 80 Daehak-ro, Buk-gu, Daegu 702-701, Korea; ByWonhwalee@gmail.com; 3 Department of Biochemistry and Cell Biology, BK21 Plus KNU Biomedical Convergence Program, School of Medicine, Kyungpook National University, Daegu 700-422, Korea

**Keywords:** antiplatelet, anticoagulant, aPTT, amidinobenzamide, nonamidinobenzamide

## Abstract

Three amidino- and ten non-amidinobenzamides were synthesized as 3-aminobenzoic acid scaffold-based anticoagulant and antiplatelet compounds. The anticoagulant activities of thirteen synthesized compounds **1**–**13**, and **2b** and **3b** as prodrugs were preliminary evaluated by screening the prolongation of activated partial thromboplastin time (aPTT) and prothrombin time (PT) *in vitro*. From the aPTT results obtained, two amidinobenzamides, *N*-(3′-amidinophenyl)-3-(thiophen-2′′-ylcarbonylamino) benzamide (**1**, 33.2 ± 0.7 s) and *N*-(4′-amidinophenyl)-3-(thiophen-2′′-ylcarbonylamino) benzamide (**2**, 43.5 ± 0.6 s) were selected to investigate the further anticoagulant and antiplatelet activities. The aPTT results of **1** (33.2 ± 0.7 s) and **2** (43.5 ± 0.6 s) were compared with heparin (62.5 ± 0.8 s) *in vitro* at 30 μM. We investigated the effect of **1** and **2** on blood anticoagulant activity (*ex vivo*) and on tail bleeding time (*in vivo*) on mice. A tail cutting/bleeding time assay revealed that both **1** and **2** prolonged bleeding time in mice at a dose of 24.1 g/mouse and above. Compounds **1** and **2** dose-dependently inhibited thrombin-catalyzed fibrin polymerization and platelet aggregation. In addition, **1** and **2** were evaluated on the inhibitory activities of thrombin and FXa as well as the generation of thrombin and FXa in human umbilical vein endothelial cells (HUVECs). Collectively, **1** and **2** possess some antiplatelet and anticoagulant activities and offer a basis for development of a novel antithrombotic product.

## 1. Introduction

Thromboembolic diseases are leading causes of cardiovascular-associated morbidity and death [1]. Prevention and treatment of arterial thrombosis in patients with cardiovascular diseases (e.g., atherosclerotic vascular disease, acute coronary syndrome) are being achieved using antiplatelet drugs [2], whereas venous thrombosis illness, such as deep vein thrombosis (DVT) and pulmonary embolism (PE), are treated with anticoagulant drugs [3,4].

Several anticoagulants, such as heparins (unfractionated and low-molecular-weight heparins) and vitamin K antagonists (e.g., warfarin), have proved to be effective in the prevention and treatment of these thrombotic diseases, but considerable shortcomings, such as inconvenient drug administration, unneglectable side effects for heparins, and extensive drug and food interactions for Vitamin K antagonists, restrict their clinical use [5]. However, fondaparinux sodium (Arixtra^®^), heparin pentasaccharide, is the first of a new class of synthetic indirect antithrombotic agents, distinct from low molecular weight heparin and heparin. Its pharmacokinetic properties allow for a simple, fixed-dose, once-daily regimen of subcutaneous injection, without the need for monitoring [6,7]. Blood clotting in response to vascular injury requires the activation of zymogens in the coagulation cascade, in which thrombin and FXa are key players. Direct FXa inhibitors such as rivaroxaban, apixaban, and edoxaban function at the convergence of the extrinsic and intrinsic coagulation pathways without the need for routine coagulation monitoring [8,9]. Thrombin inhibitors, such as dabigatran etexilate (dabigatran) and argatroban, play an important role in the anticoagulation process [10,11].

In addition, aspirin, which irreversibly inhibits cyclooxygenase 1-mediated transformation of arachidonic acid to thromboxane A_2_ (TXA_2_), and the P_2_Y_12_ antagonists, clopidogrel and prasugrel, which selectively and irreversibly bind to the P_2_Y_12_ ADP receptor, are routinely used as antiplatelet agents [12,13]. However, there are still some serious limitations to these agents, which include weak inhibition of platelet function and bleeding events of aspirin [14,15] and slow onset of action and bleeding events of clopidogrel [16,17].

Furthermore, a combination of drugs causing antithrombosis with different antiplatelet mechanisms was reported to improve clinical efficiency and safety. For instance, aspirin and clopidogrel [18], aspirin plus dipyridamole [19], or aspirin plus glycoprotein IIb/IIIa-receptor inhibitors [20], are successfully used in stroke prevention. Moreover, enhanced antiplatelet activity has been described for the combined administration of TXA_2_ synthase inhibitor (Dazoxiben) plus TXA_2_ receptor antagonist (Sulotroban) in experimental assays [21].

In addition, the combined use of anticoagulant and antiplatelet agents showed additional benefits over anticoagulants alone in patients with prosthetic heart valves [22] and in patients with atrial fibrillation [23]. A number of clinical data have shown that the combination of antiplatelet agents (e.g., aspirin or clopidogrel) and anticoagulant agents (e.g., warfarin or heparin) has an additive effect by suppressing both blood coagulation and platelet aggregation, as well as combinations of aspirin plus warfarin [24], aspirin plus heparin [25], warfarin plus clopidogrel [26], direct thrombin inhibitor plus GpIIb/IIIa antagonists [27], ADP inhibitor plus FXa inhibitors [28], and warfarin plus amiodarone [29]. However, because such combinations may also increase the risk of bleeding, these will require careful monitoring in large clinical trials of patients, with particular attention to the elderly [20].

Even though, compounds combining anticoagulant and platelet antiaggregating activities in the same molecule are believed to be promising drugs [28]. Herein, we described the synthesis of amidino- and non-amidinobenzamides as 3-aminobenzoic acid scaffold-based anticoagulant and antiplatelet compounds. The antiplatelet effects on thrombin, U46619, and thrombin-catalyzed fibrin polymerization, as well as the regulation of clotting time (aPTT and PT), were determined. Additionally, the anticoagulant activities of synthesized compounds on direct thrombin and FXa inhibition, the generation of thrombin, and FXa were evaluated.

## 2. Results and Discussion

### 2.1. Chemistry

**Scheme 1:**
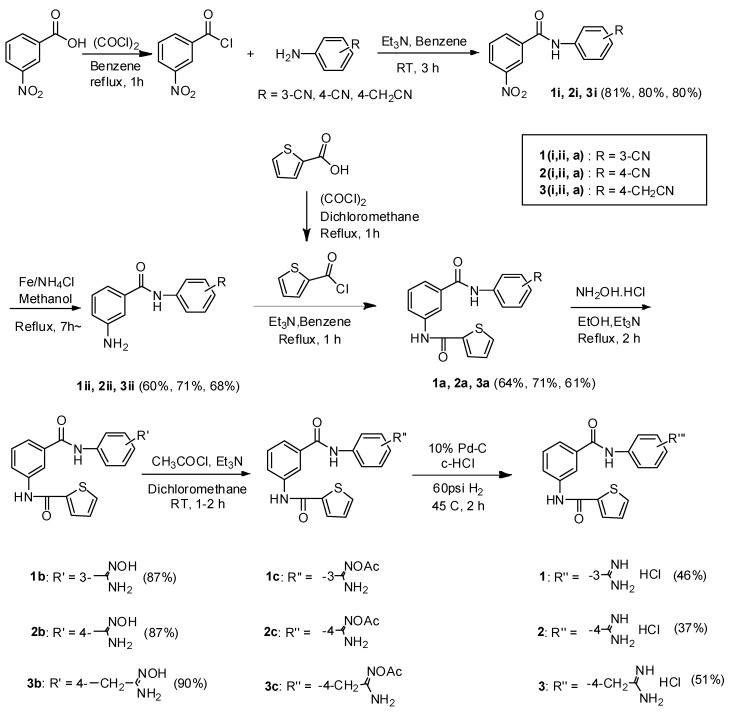
Synthetic methods of amidinobenzamides **1**–**3**.

Using diversely substituted 3-aminobenzoic acid as the central scaffold, a series of FXa inhibitors was patented [30]. The FXa inhibitors containing a carboxyl group at the central phenyl ring were reported. Considering the potential of 3-aminobenzoic acid as an anticoagulant agent, we tried to synthesize 1,3-diamide derivatives containing the amidino- and nonamidino group from 3-aminobenzoic acid. Scheme 1 illustrates the synthesis of amidinobenzamides **1**–**3**.

The first side chain was introduced through acylation of 3-nitrobenzoyl chloride with 3-cyano-, 4-cyano-, and 4-cyanomethylaniline to give **1i**–**3i**. The nitro group of these 3-nitrobenzamides was reduced by NH_4_Cl and Fe to give amines **1ii**–**3ii**. An acylation with thiophene-2-carbonyl chloride provided the *N*-(thiophene-2-carbonyl) 3- or 4-cyanophenylbenzamides **1a** and **2a**, and the *N*-(thiophene-2-carbonyl) 4-cyanobenzylbenzamid **3a**, which were converted to amidoximes **1b**–**3b** by treatment with hydroxylamine·HCl and triet-hylamine. The N–O bond of amidoximes **1b**–**3b** was converted to amidinium chloride **1**–**3** via the *O*-acetyl amidoximes **1c**–**3c** and catalytic hydrogenated over 10% Pd/C in the presence of c-HCl at 60 psi, 45 °C for 2 h. In the ^1^H-NMR spectra, the four proton peaks of amidinium chloride in **1** were identified as one broad singlet at 9.45 ppm, and otherwise the four proton peaks of amidinium chloride in **2** and **3** were identified, respectively, as two broad singlets at 9.17 and 9.36 ppm and at 8.83 and 9.28 ppm in the ^1^H-NMR spectra. Two NH proton peaks of compound **2** were confirmed by observing the correlation of NH (10.78 ppm) and CO (164.9 ppm) of the –NHCO group and of NH (10.63 ppm) and CO (166.1 ppm) of the –CONH group in the HMBC spectrum, respectively.

**Scheme 2:**
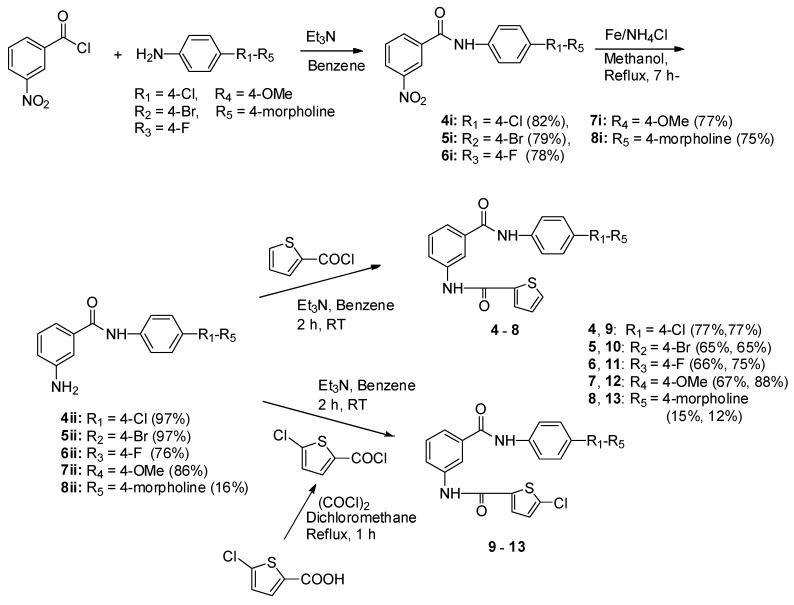
Synthetic methods of non-amidinobenzamides **4**–**13**.

Instead of a basic amidine group, neutral substituents which improve oral bioavailability and are still capable of maintaining hydrogen bonding with the enzyme are required for FXa and thrombin inhibitors. Anilines containing 4-chloro-, 4-bromo-, 4-fluoro-, 4-methoxy-, and 4-morpholine as a neutral substituent were coupled with 3-nitrobenzoyl chloride to synthesize the corresponding 3-nitrobenzamide derivatives **4i**–**8i**, respectively, in Scheme 2. The reduction of the nitro group of **4i**–**8i** with NH_4_Cl and Fe produced the 3-aminobenzamides **4ii**–**8ii**. The second acylation of **4ii**–**8ii** was performed by treatment with thiophene-2-carbonyl chloride to give **4**–**8** and also 5-chloro-thiophene-2-carbonyl chloride to give **9**–**13**, respectively.

### 2.2. Biology

#### 2.2.1. Effects of Synthesized Compounds on Aptt, PT, and Tail Bleeding Time

The anticoagulant effects of fifteen synthesized compounds (**2b**, **3b,** and **1**–**13**) were screened in aPTT and PT assays using human plasma at a concentration of 30 μg/mL and are summarized in Table 1. From these aPTT results, *N*-(3′-amidinophenyl)-3-(thiophen-2′′-ylcarbonylamino) benzamide **1** and *N*-(4′-amidinophenyl)-3-(thiophen-2′′-ylcarbonylmino) benzamide **2** were selected as target for the further anticoagulant and antiplatelet experiments. Both aPTT and PT were not prolonged by amidoximes **2b** and **3b** as prodrugs of **1** and **2** and non-amidinobenzamides **4**–**13** at concentrations 30 μg/mL. Most of the amidine-type compounds were found to be insufficiently absorbed when administered orally, because of strongly basic amidine groups [11]. Therefore, the trend in synthetic studies seems to be shifted to non-amidine-type compounds containing weak basic groups. However, our non-amidine compounds did not show prolongation in aPTT and PT. The amidine group seems to be needed for the anticoagulant activity.

We investigated the effect of **1** and **2** on anticoagulant activities (*in vitro* and *ex vivo*) and on tail bleeding time (*in vivo*) on mice. As shown in Table 2, aPTT in the vehicle-treated group was 23.6 ± 0.6 s (mean ± SEM, *n* = 5) and in amidines **1**, **2** and heparin, aPTT increased 33.2 ± 0.7 s, 43.5 ± 0.6 s and 62.5 ± 0.8 s at dose 30 μM, respectively. Although the *in vitro* anticoagulant activities of **1** and **2** were weaker than those of heparin, aPTT was significantly prolonged by **1** and **2** at concentrations 20 μM and above, as compared to the vesicle group, while no obvious increase in PT was found. Noting that a prolongation of aPTT suggests the inhibition of the intrinsic and/or common coagulation pathway, and a PT prolongation suggests inhibition of the extrinsic and/or common pathway, obtained results in this study showing prolongation of aPTT of compounds **1** and **2** suggest inhibition of the intrinsic pathway and/or common pathway by compounds **1** and **2**.

To confirm *in vitro* anticoagulant activity, *in vivo* tail bleeding times were evaluated. The average circulating blood volume for mice is 72 mL/kg [31]. Because the average weight of the mouse used is 27 g, the molecular weight of **1** or **2** is 400.88, and the average blood volume is 2 mL, the amount of synthesized compounds (24.1, 32.1, or 40.1 μg/mouse) injected yielded a maximum concentration of 30, 40, or 50 μM in the peripheral blood. As shown in Table 3, tail bleeding times were significantly prolonged by compounds **1** and **2** at concentrations 24.1 μg/mouse and above, as compared to the controls.

aPTT values were also significantly prolonged by **1** and **2** at a concentration of 24.1 μg/mouse and above *ex vivo* clotting times, while no obvious increase in PT values was found (Table 4). Collectively, aPTT (*in vitro* and *ex vivo*) and tail bleeding time (*in vivo*) on the mice of **2** were longer than those of **1**, suggesting that the *para-*amidine group is more efficient than the *meta*-amidine group on anticoagulant activity.

#### 2.2.2. Effects of **1** or **2** on Thrombin-Catalyzed Fibrin Polymerization and Platelet Aggregation

**Figure 1:**
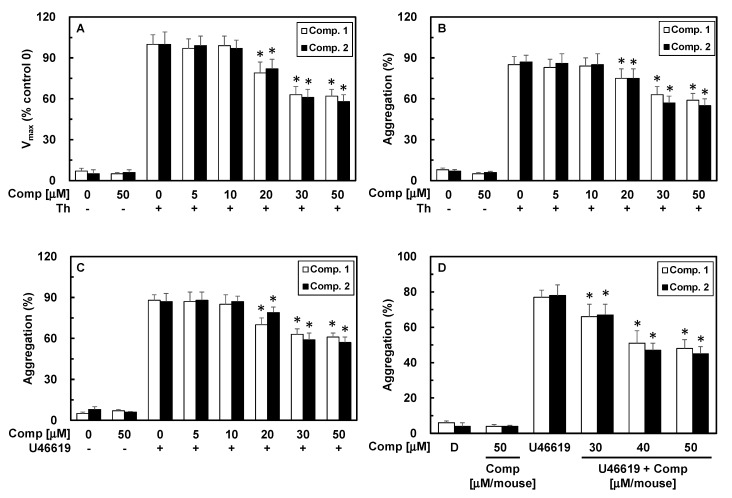
Effects of **1** or **2** on fibrin polymerization in human plasma. (**A**) Thrombin-catalyzed fibrin polymerization at the indicated concentrations of **1** or **2** was monitored using a catalytic assay, as described in the “Experimental Section”. The results are V_max_ values expressed as percentages versus controls; (**B**) Effect of **1** or **2** on mouse platelet aggregation induced by 3 U/mL thrombin; (**C**) The effect of each compound on human platelet aggregation induced by 2 mM U46619; (**D**) The indicated amount of each compound concentration in DMSO was injected intravenously. The effects of each compound on mouse platelet aggregation induced by 2 μM U46619 were monitored *ex vivo*. D = 0.2% DMSO is the vehicle control. Data represent the mean ± SEM of three independent experiments performed in triplicate. * *p* < 0.05 *vs.* Th or U46619 alone.

The effects of **1** or **2** on thrombin-catalyzed fibrin polymerization in human plasma were monitored as changes in absorbance at 360 nm, as described in the Experimental Section. The results, shown in Figure 1A, demonstrate that incubation of human plasma with **1** or **2** resulted in a significant decrease in the maximum rate of fibrin polymerization (Figure 1A). To eliminate the effect of sample pH, all dilutions were performed using 50 mM TBS (pH 7.4). We also evaluated the effect of the same volume of DMSO on human plasma; however, coagulation properties were unaffected. To confirm the antiplatelet activities of compounds **1** and **2**, a thrombin-catalyzed platelet aggregation assay was performed. As shown in Figure 1B, treatment with compounds **1** or **2** resulted in significantly inhibited mouse platelet aggregation induced by thrombin (final concentration: 3 U/mL) in a concentration-dependent manner. In order to exclude the possibility that the decrease of polymerization could be due to a direct effect on thrombin leading to a decrease in fibrin generation, rather than polymerization of fibrin formed, a reptilase-catalyzed polymerization assay was performed. Results showed that **1** and **2** induced a significant decrease in reptilase-catalyzed polymerization (data not shown). To confirm the antiplatelet activities of compounds **1** or **2**, a U46619-(a stable thromboxane A2 analog/aggregation agonist) catalyzed platelet aggregation assay was performed. The thromboxane A2 pathway is a major contributor to the amplification of the initial platelet activation process. As shown in Figure 1C, treatment with compounds **1** or **2** significantly inhibited human platelet aggregation induced by U46619 (final concentration: 2 μM) in a concentration-dependent manner. These *in vitro* results were confirmed in an *ex vivo* platelet aggregation assay (i.v. injection, Figure 1D). As shown in Figure 1D, treatment with **1** or **2** resulted in significantly inhibited mouse platelet aggregation induced by U46619 (final concentration: 2 μM) in a concentration-dependent manner [32,33]. So far, most of the amidine-type compounds have been reported as FXa inhibitors, and these amidine derivatives **1** and **2** also exhibited potential as thromboxane A2 receptor antagonists.

#### 2.2.3. Thrombin and Factor Xa (FXa) Activity

**Figure 2:**
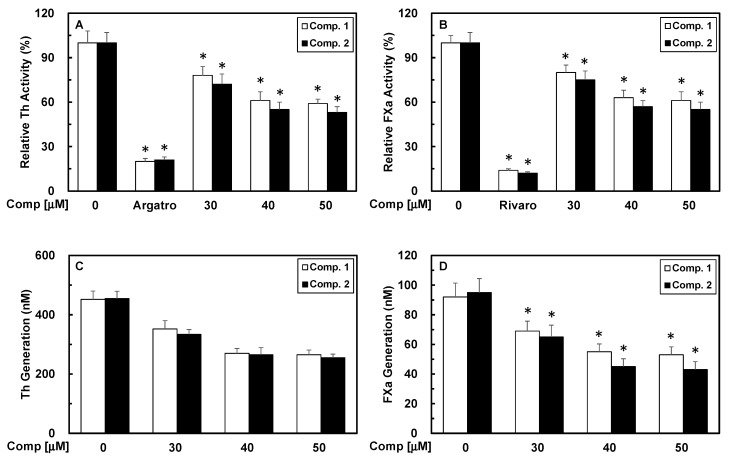
Effects of **1** or **2** on inactivation and production of thrombin and factor Xa. (**A**) Inhibition of thrombin (Th) by **1** or **2** was measured using a chromogenic assay, as described in the “Experimental Section”; (**B**) Inhibition of factor Xa (FXa) by **1** or **2** was monitored using a chromogenic assay, as described in the “Experimental Section”. Argatroban (A) or rivaroxaban (B) was used as a positive control; (**C**) HUVEC monolayers were pre-incubated with FVa (100 pM) and FXa (1 nM) for 10 min with the indicated concentrations of **1** or **2**. Prothrombin was added to a final concentration of 1 M and prothrombin activation was determined 30 min later, as described in the “Experimental Section”; (**D**) HUVECs were pre-incubated with the indicated concentrations of **1** or **2** for 10 min. After TNF-(10 ng/mL for 6 h) stimulated HUVECs were incubated with FVIIa (10 nM) and FX (175 nM), FXa production was determined, as described in the “Experimental Section”. D = 0.2% DMSO is the vehicle control. * *p* < 0.05 *vs*. 0.

In order to determine the underlying mechanism whereby **1** and **2** mediated inhibition of coagulation, the effect of **1** and **2** on the activities of thrombin and FXa were measured. As shown in Figure 2A, we also investigated the effects of **1** and **2** on the activity of thrombin. Compounds **1** and **2** dose-dependently inhibited the activity of thrombin. The direct thrombin inhibitor, argatroban was used as a positive control. In addition, treatment with **1** and **2** resulted in a dose-dependent inhibition of amidolytic activity of FXa, indicating direct inhibition of FXa activity. Ribaroxaban, a direct FXa inhibitor, was used as a positive control (Figure 2B).

#### 2.2.4. Thrombin and Factor Xa (FXa) Generation

In a previous study, Sugo *et al*. [34] reported that endothelial cells are able to support prothrombin activation by FXa. In the current study, pre-incubation of HUVECs with FVa and FXa in the presence of CaCl_2_ prior to addition of prothrombin resulted in production of thrombin (Figure 2C). According to Rao *et al*. [35], the endothelium provides the functional equivalent of pro-coagulant phospholipids and supports activation of FXa, and, in TNF-α stimulated HUVECs, activation of FX by FVIIa occurred in a tissue factor (TF) expression-dependent manner. Thus, we investigated the effects of **1** and **2** on activation of FX by FVIIa. Pre-incubation with **1** and **2** resulted in dose-dependent inhibition of FX activation by FVIIa (Figure 2D). Therefore, these results suggested that **1** and **2** can inhibit production of thrombin and FXa.

#### 2.2.5. Cellular Viability

**Figure 3:**
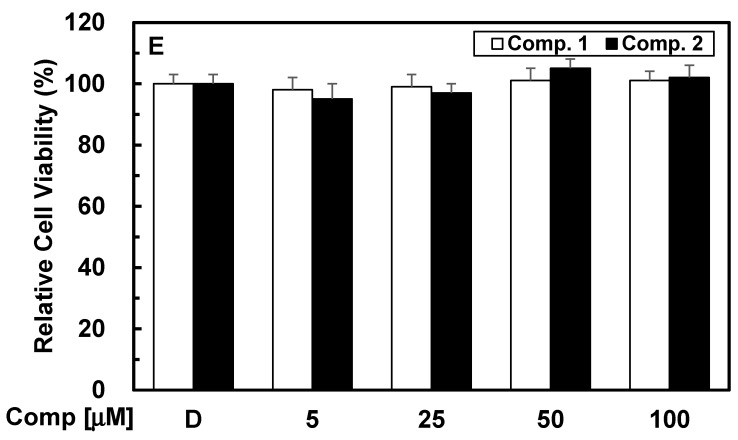
Effects of **1** or **2** on cellular viability was measured by MTT assay. D = 0.2% DMSO is the vehicle control.

To determine the cellular viability of compounds **1** and **2**, the cellular viability assay (MTT assay) was performed in HUVECs treated with **1** and **2** for 24 h. Both **1** and **2** did not affect cell viability at concentration up to 100 μM (Figure 3).

## 3. Experimental Section

### 3.1. Chemistry

#### 3.1.1. Reagents and Instruments

All non-aqueous reactions were performed under a dry atmosphere of nitrogen. The commercial reagents were purchased from Sigma-Aldrich (St. Louis, MO, USA) or TCI (Tokyo, Japan). Solvents were purified and dried prior to use. Melting points were measured on Thomas-Hoover melting point apparatus (Thomas Scientific, Swedesboro, NJ, USA) and not corrected. ^1^H, ^13^C-NMR and HMBC spectra were taken on a Varian 400 MHz spectrometer (Thomas Scientific, Swedesboro, NJ, USA) in DMSO-*d*_6_. Chemical shifts (δ) are in parts per million (ppm) relative to tetramethylsilane, and coupling constants (*J*) are in Hertz. DIP-MS (EI) was obtained on an Agilent 7890A-5975C GC/MSD (Agilent Technologies, Santa Clara, CA, USA). GC/MS (EI) was obtained on a SHIMADZU QP 2010 model (Shimadzu, Kyoto, Japan) and FAB-MS was obtained on a JEOL JMS-700 Mstation (JEOL, Tokyo, Japan). Fraction collection was performed on an EYELA fraction collector DC-1500 (Tokyo Rikakikai, Tokyo, Japan). An analytical TLC was performed on pre-coated silica gel 60 F_254_ plates (Merck, Kenilworth, NJ, USA). Solvent systems for TLC were ethyl acetate/*n*-hexane mixtures and 10% methanol in dichloromethane. Column chromatography was carried out on Merck silica gel 9385 (Merck, Kenilworth, NJ, USA) (230–400 mesh).

#### 3.1.2. General Experimental Procedures of **1i**–**3i**

To a stirred solution of 3-nitrobenzoic acid (35.90 mmol) in anhydrous benzene (50 mL) was dropwise added: oxalyl chloride (46.67 mmol) and then triethylamine (39.49 mmol) at room temperature. The reaction mixture was refluxed for 1 h and the solvent and the unreacted oxalyl chloride were evaporated off under reduced pressure and the acid chloride was used without purification. To a solution of 3-aminobenzonitrile, 4-aminobenzonitrile, and 4-aminobenzyl cyanide (8.62 mmol) in anhydrous benzene (50 mL) was added: acyl chloride (10.78 mmol) and triethylamine (8.62 mmol) at room temperature and stirred for 3 h. To the reaction mixture, water was added and extracted with ethyl acetate (50 mL × 3), dried with anhydrous magnesium sulfate and filtrated. The filtrate was evaporated under reduced pressure to give crude compound, which was recrystallized with ethyl acetate and *n-*hexane to give a pure white or pale yellow compound, respectively.

*3-Nitro-N-(3′-cyanophenyl) benzamide* (**1i**)*.* Yield: 81%; m.p.: 195–196 °C; ^1^H-NMR (400 MHz, DMSO-*d*_6_) δ: 7.62 (2H, d, *J* = 8.0 Hz, H-6′,4′), 7.86 (1H, t, *J* = 8.0 Hz, H-5′), 8.06 (1H, t, *J* = 8.0 Hz, H-5), 8.26 (1H, s, H-2′), 8.41 (1H, ddd, *J* = 7.8, 1.5, 1.1 Hz, H-6), 8.47 (1H, ddd, *J* = 8.2, 2.3, 1.0 Hz, H-4), 8.81 (1H, t, *J* = 2.0 Hz, H-2), 10.88 (1H, s, CONH); ^13^C-NMR (100 MHz, DMSO-*d*_6_) δ: 111.5 (C-3′), 118.6 (C≡N), 122.5 (C-6′), 123.3 (C-2), 125.1(C-5′), 126.5 (C-4), 127.6 (C-2′), 130.3 (C-4′), 134.3 (C-6), 135.7 (C-1), 139.5 (C-1′), 147.8 (C-3), 163.8 (C=O); GC-MS (EI) *m*/*z*: 267 [M]^+^.

*3-Nitro-N-(4′-cyanophenyl) benzamide* (**2i**). Yield: 80%; m.p.: 225–226 °C; ^1^H-NMR (400 MHz, DMSO-*d*_6_) δ: 7.85 (2H, d, *J* = 8.0 Hz, H-3′,5′), 7.86 (1H, t, *J* = 8.0 Hz, H-5), 7.99 (2H, d, *J* = 8.0 Hz, H-2′,6′), 8.41 (1H, d, *J* = 8.0 Hz, H-6), 8.46 (1H, ddd, *J* = 8.2, 2.3, 1.0 Hz, H-4), 8.67 (1H, t, *J* = 2.0 Hz, H-2), 10.96 (1H, s, CONH); ^13^C-NMR (100 MHz, DMSO-*d*_6_) δ: 106.6 (C-4′), 119.6 (C≡N), 121.1 (C-2′,6′), 123.3 (C-2), 127.2 (C-4), 131.0 (C-5), 133.9 (C-3′,5′), 135.0 (C-6), 136.4 (C-1), 143.7 (C-1′), 148.4 (C-3), 164.7 (C=O); GC-MS (EI) *m*/*z:* 267 [M]^+^.

*3-Nitro-N-(4′-cyanomethylphenyl) benzamide* (**3i**). Yield: 80%; m.p.: 197–198 °C; ^1^H-NMR (400 MHz, DMSO-*d*_6_) δ: 3.97 (2H, s, CH_2_), 7.31 (2H, d, *J* = 8.8 Hz, H-3′,5′), 7.76 (2H, d, *J* = 8.8 Hz, H-2′,6′), 7.80 (1H, t, *J* = 8.0 Hz, H-5), 8.36 (1H, d, *J* = 8.0 Hz, H-6), 8.40 (1H, ddd, *J* = 8.2, 2.3, 1.0 Hz, H-4) 8.75 (1H, t, *J* = 2.0 Hz, H-2), 10.60 (1H, s, CONH); ^13^C-NMR (100 MHz, DMSO-*d*_6_) δ: 21.9 (CH_2_), 119.3 (C≡N), 121.0 (C-2′,6′), 122.4 (C-2), 126.2 (C-4′), 126.7 (C-4), 128.4 (C-3′,5′), 130.2 (C-5), 134.2 (C-6), 136.1 (C-1), 138.1 (C-1′), 147.7 (C-3), 163.3 (C=O); GC-MS (EI) *m*/*z:* 281 [M]^+^.

#### 3.1.3. General Experimental Procedures of **1ii**–**3ii**

To a solution of **1i**–**3i** (1.79 mmol) in methanol (20 mL) was added: ammonium chloride (double amount of **1i**–**3i**) and iron powder (3.58 mmol), and the reaction mixture was refluxed for 7 h. To the reaction mixture, ice water was added and rotary evaporated to remove methanol under reduced pressure. The aqueous residue was extracted with dichloromethane (30 mL × 3) and the organic phase was dried with anhydrous MgSO_4_, filtrated, and evaporated to give the crude solid, which was recrystallized with ethyl acetate and *n*-hexane to give a pure white or pale-yellow solid.

*3-Amino-N-(3′-cyanophenyl) benzamide* (**1ii**). Yield: 60%; m.p.: 166–168 °C; ^1^H-NMR (400 MHz, DMSO-*d*_6_) δ: 5.35 (2H, s, NH_2_), 6.77 (1H, ddd, *J* = 8.2, 2.3, 1.0 Hz, H-4), 7.07 (1H, d, *J* = 7.6 Hz, H-6), 7.10 (1H, t, *J* = 1.9 Hz, H-2), 7.17 (1H, t, *J* = 7.6 Hz, H-5), 7.54-7.58 (2H, m, H-4′,5′), 8.04 (1H, dt, *J* = 7.2, 2.2 Hz, H-6′), 8.24 (1H, s, H-2′), 10.39 (1H, s, CONH); ^13^C-NMR (100 MHz, DMSO-*d*_6_) δ: 111.4 (C-3′), 112.9 (C-2), 114.7 (C-6), 117.1 (C-4), 118.8 (C≡N), 122.8 (C-6′), 124.7 (C-5′), 126.9 (C-2′), 128.9 (C-5), 130.1 (C-4′), 135.2 (C-1), 140.2 (C-1′), 148.9 (C-3), 166.8 (C=O); GC-MS (EI) *m*/*z:* 237 [M]^+^.

*3-Amino-N-(4′-cyanophenyl) benzamide* (**2ii**). Yield: 71%; m.p.: 182–183 °C; ^1^H-NMR (400 MHz, DMSO-*d*_6_) δ: 5.36 (2H, s, NH_2_), 6.77 (1H, ddd, *J* = 8.0, 2.0, 1.2 Hz, H-4), 7.05 (1H, d, *J* = 7.6 Hz, H-6), 7.07 (1H, t, *J* = 1.9 Hz, H-2), 7.17 (1H, t, *J* = 8.0 Hz, H-5), 7.80 (2H, d, *J* = 8.0 Hz, H-3′,5′), 7.98 (2H, d, *J* = 8.0 Hz, H-2′,6′), 10.48 (1H, s, CONH); ^13^C-NMR (100 MHz, DMSO-*d*_6_) δ: 105.0 (C-4′), 112.9 (C-2), 114.8 (C-6), 117.2 (C-4), 119.1 (C≡N), 120.0 (C-2′,6′), 128.9 (C-5), 133.1 (C-3′,5′), 135.3 (C-1), 143.7 (C-1′), 148.9 (C-3), 167.0 (C=O); GC-MS (EI) *m*/*z:* 237 [M]^+^.

*3-Amino-N-(4′-cyanomethylphenyl) benzamide* (**3ii**). Yield: 68%; m.p.: 173–174 °C; ^1^H-NMR (400 MHz, DMSO-*d*_6_) δ: 3.98 (2H, s, CH_2_), 5.31 (2H, s, NH_2_), 6.74 (1H, ddd, *J* = 7.8, 2.2, 1.2 Hz, H-4), 7.05 (1H, d, *J* = 7.6 Hz, H-6), 7.09 (1H, t, *J* = 1.9 Hz, H-2), 7.14 (1H, t, *J* = 8.0 Hz, H-5), 7.30 (2H, d, *J* = 8.0 Hz, H-3′,5′), 7.78 (2H, d, *J* = 8.0 Hz, H-2′,6′), 10.13 (1H, s, CONH); ^13^C-NMR (100 MHz, DMSO-*d*_6_) δ: 21.9 (CH_2_), 112.9 (C-2), 114.7 (C-6), 116.8 (C-4), 119.4 (C≡N), 120.6 (C-2′,6′), 125.9 (C-4′), 128.3 (C-3′,5′), 128.7 (C-5), 135.8 (C-1), 138.8 (C-1′), 148.8 (C-3), 166.4 (C=O); GC-MS (EI) *m*/*z:* 251 [M]^+^.

#### 3.1.4. General Experimental Procedures of **1a**–**3a**

To a solution of thiophene-2-carboxylic acid (7.8 mmol) in anhydrous dichloromethane (40 mL) was added: triethylamine (8.6 mmol) and dropwise added oxalyl chloride (10.1 mmol) at room temperature. The reaction mixture was refluxed for 1 h and rotary evaporated to remove dichloromethane and oxalyl chloride, which was used next reaction without purification. To a solution of **1ii**–**3ii** (1.48 mmol) in anhydrous benzene (20 mL) was added: triethylamine (1.48 mmol) and thiophene-2-carbonyl chloride (1.85 mmol) at room temperature and the reaction mixture was refluxed for 1 h. To the reaction mixture, water was added and extracted with ethyl acetate (50 mL × 3), dried with anhydrous MgSO_4,_ and filtrated. The filtrate was evaporated under reduced pressure to give a crude compound, which was recrystallized with ethyl acetate and *n-*hexane to give a pure white or pale yellow compound, respectively.

*N-(3′-Cyanophenyl)-3-(thiophen-2′′-ylcarbonylamino) benzamide* (**1a**). Yield: 64%; m.p.: 201–202 °C; ^1^H-NMR (400 MHz, DMSO-*d*_6_) δ: 7.25 (1H, dd, *J* = 5.0, 4.0 Hz, H-4′′), 7.54 (1H, t, *J* = 8.0 Hz, H-5), 7.58-7.60 (2H, m, H-4′,5′), 7.71 (1H, d, *J* = 6.8 Hz, H-6), 7.89 (1H, dd, *J* = 5.0, 1.0 Hz, H-5′′), 8.03 (1H, dd, *J* = 8.0, 0.8 Hz, H-3′′), 8.07-8.09 (2H, m, H-4,6′), 8.26 (1H, t, *J* = 1.2 Hz, H-2′), 8.28 (1H, t, *J* = 1.8 Hz, H-2), 10.47 (1H, s, NHCO), 10.61 (1H, s, CONH); ^13^C-NMR (100 MHz, DMSO-*d*_6_) δ: 111.5 (C-3′), 118.7 (C≡N), 119.8 (C-2), 122.7 (C-6), 123.0 (C-4), 123.6 (C-6′), 124.9 (C-5′), 127.2 (C-2′), 128.2 (C-5), 128.9 (C-4′′), 129.4 (C-3′′), 130.2 (C-4′), 132.2 (C-5′′), 135.0 (C-1), 139.0 (C-3), 139.7 (C-2′′), 140.0 (C-1′), 160.1 (CONH), 165.9 (NHCO); DIP-MS (EI) *m*/*z:* 347 [M]^+^.

*N-(4′-Cyanophenyl)-3-(thiophen-2′′-ylcarbonylamino) benzamide* (**2a**). Yield: 71%; m.p.: 214–216 °C; ^1^H-NMR (400 MHz, DMSO-*d*_6_) δ: 7.25 (1H, dd, *J* = 5.0, 4.0 Hz, H-4′′), 7.54 (1H, t, *J* = 8.0 Hz, H-5), 7.71 (1H, d, *J* = 6.8 Hz, H-6), 7.83 (2H, d, *J* = 8.0 Hz, H-3′,5′), 7.88 (1H, dd, *J* = 4.0, 0.8 Hz, H-5′′), 8.00 (2H, d, *J* = 8.0 Hz, H-2′,6′), 8.03 (1H, ddd, *J* = 8.0, 2.3, 1.0 Hz, H-4), 8.09 (1H, dd, *J* = 4.0, 0.8 Hz, H-3′′), 8.28 (1H, t, *J* = 1.9 Hz, H-2), 10.47 (1H, s, NHCO), 10.70 (1H, s, CONH); ^13^C-NMR (100 MHz, DMSO-*d*_6_) δ: 105.4 (C-4*′*), 119.2 (C≡N), 119.9 (C-2), 120.2 (C-2′,6′), 122.8 (C-6), 123.7 (C-4), 128.2 (C-5), 128.9 (C-4′′), 129.4 (C-3′′), 132.2 (C-5′′), 133.1 (C-3′,5′), 135.0 (C-1), 139.1 (C-3), 139.7 (C-2′′), 143.5 (C-1′), 160.1 (CONH), 166.1 (NHCO); DIP-MS (EI) *m*/*z:* 347 [M]^+^.

*N-(4′-Cyanomethylphenyl)-2-(thiophen-2′′-ylcarbonylamino) benzamide* (**3a**). Yield: 61%; m.p.: 202–203 °C; ^1^H-NMR (400 MHz, DMSO-*d*_6_) δ: 4.01 (2H, s, CH_2_), 7.25 (1H, dd, *J* = 5.0, 4.0 Hz, H-4′′), 7.34 (2H, d, *J* = 8.0 Hz, H-3′,5′), 7.52 (1H, t, *J* = 8.0 Hz, H-5), 7.70 (1H, d, *J* = 8.0 Hz, H-6), 7.81 (2H, d, *J* = 8.0 Hz, H-2′,6′), 7.88 (1H, dd, *J* = 4.0, 0.8 Hz, H-5′′), 8.01 (1H, ddd, *J* = 8.0, 2.3, 1.0 Hz, H-4), 8.08 (1H, dd, *J* = 4.0, 0.8 Hz, H-3′′), 8.26 (1H, t, *J* = 2.0 Hz, H-2), 10.36 (1H, s, NHCO), 10.43 (1H, s, CONH); ^13^C-NMR (100 MHz, DMSO-*d*_6_) δ: 21.9 (CH_2_), 119.3 (C≡N), 119.8 (C-2), 120.7 (C-2′,6′), 122.7 (C-6), 123.3 (C-4), 126.2 (C-4′), 128.1 (C-5), 128.4 (C-3′,5′), 128.7 (C-4′′), 129.3 (C-3′′), 132.1 (C-5′′), 135.5 (C-1), 138.6 (C-3), 138.9 (C-1′), 139.7 (C-2′′), 160.0 (CONH), 165.5 (NHCO); DIP-MS (EI) *m*/*z:* 361 [M]^+^.

#### 3.1.5. General Experimental Procedures of **1b**–**31ii**–**3ii**

To a suspension of intermediate **1a**–**3a** (0.86 mmol) in absolute ethanol (25 mL) and 1,4-dioxane (5 mL) was added: triethylamine (2.59 mmol) and refluxed to dissolve. The reaction mixture was added with hydroxylamine·HCl (3.45 mmol) and refluxed for 4–6 h. The mixture was evaporated to remove the ethanol and ice water was poured into the residue. The resulting precipitate was filtrated, washed with water, and dried under reduced pressure to give a pure white or pale yellow solid.

*N-(3′-Amidoximephenyl)-3-(thiophen-2′′-ylcarbonylamino) benzamide* (**1b**). Yield: 87%; m.p.: 234–235 °C; ^1^H-NMR (400MHz, DMSO-*d*_6_) δ: 5.75 (2H, s, NH_2_), 7.24 (1H, dd, *J* = 4.9, 3.8 Hz, H-4′′), 7.34–7.38 (2H, m, H-5, 4′), 7.51 (1H, t, *J* = 7.9 Hz, H-5′), 7.71 (1H, d, *J* = 7.8 Hz, H-4), 7.80 (1H, d, *J* = 7.6 Hz, H-6), 7.88 (1H, dd, *J* = 5.0, 0.8 Hz, H-5′′), 8.01 (1H, dd, *J* = 8.0, 1.2 Hz, H-6′), 8.05–8.10 (2H, m, H-2′,3′′), 8.27 (1H, t, *J* = 2.0 Hz, H-2), 9.62 (1H, s, NOH), 10.35 (1H, s, NHCO), 10.47 (1H, s, CONH); ^13^C-NMR (100 MHz, DMSO-*d*_6_) δ: 118.5 (C-2), 120.6 (C-2′), 121.6 (C-6′), 121.7 (C-6), 123.4 (C-4′), 124.0 (C-4), 128.8 (C-5), 128.9 (C-4′′), 129.4 (C-3′′), 130.0 (C-5′), 132.8 (C-5′′), 134.6 (C-1), 136.2 (C-1′), 139.6 (C-2′′), 140.5 (C-3), 151.6 (C=NOH), 160.7 (CONH), 166.1 (NHCO); FAB-MS *m*/*z:* 363 [M + 1 − H_2_O]^+^, 380 [M]^+^, 381 [M + 1]^+^, 403 [M + Na]^+^.

*N-(4′-Amidoximephenyl)-3-(thiophen-2′′-ylcarbonylamino) benzamide* (**2b**). Yield: 87%; m.p.: 241–242 °C; ^1^H-NMR (400 MHz, DMSO-*d*_6_) δ: 5.76 (2H, s, NH_2_), 7.25 (1H, dd, *J* = 5.0, 4.0 Hz, H-4′′), 7.52 (1H, t, *J* = 8.0 Hz, H-5), 7.66 (2H, d, *J* = 8.0 Hz, H-3′,5′), 7.70 (1H, d, *J* = 7.6 Hz, H-6), 7.78 (2H, d, *J* = 8.0 Hz, H-2′,6′), 7.88 (1H, dd, *J* = 4.0, 0.8 Hz, H-5′′), 8.01 (1H, d, *J* = 7.6, Hz, H-4), 8.08 (1H, dd, *J* = 8.0, 0.8 Hz, H-3′′), 8.25 (1H, t, *J* = 2.0 Hz, H-2), 9.55 (1H, s, NOH), 10.37 (1H, s, NHCO), 10.44 (1H, s, CONH); ^13^C-NMR (100 MHz, DMSO-*d*_6_) δ: 119.6 (C-2′,6′), 119.8 (C-2), 122.7 (C-6), 123.3 (C-4), 125.7 (C-3′,5′), 128.2 (C-5), 128.7 (C-4′), 128.9 (C-4′′), 129.3 (C-3′′), 132.1 (C-5′′), 135.5 (C-1), 139.9 (C-3), 139.6 (C-1′), 139.7 (C-2′′), 150.5 (C=NOH), 160.0 (CONH), 165.5 (NHCO); FAB-MS *m*/*z:* 363 [M + 1 − H2O]^+^, 380 [M]^+^, 381 [M + 1]^+^, 403 [M + Na]^+^.

*N-(4′-Amidoximemethylphenyl)-3-(thiophen-2′′-ylcarbonylamino) benzamide* (**3b**). Yield: 90%; m.p.: 164–165 °C; ^1^H-NMR (400 MHz, DMSO-*d*_6_) δ: 3.23 (2H, s, CH_2_), 5.37 (2H, s, NH_2_), 7.19–7.26 (3H, m, H-3′,5′,4′′), 7.50 (1H, t, *J* = 7.9 Hz, H-5), 7.67–7.69 (3H, m, H-2′,6′,4), 7.88 (1H, d, *J* = 4.9 Hz, H-5′′), 8.01 (1H, d, *J* = 8.0 Hz, H-6), 8.10 (1H, d, *J* = 3.5 Hz, H-3′′), 8.26 (1H, s, H-2), 8.88 (1H, s, NOH), 10.29 (1H, s, NHCO), 10.50 (1H, s, CONH); ^13^C-NMR (100 MHz, DMSO-*d*_6_) δ: 37.4 (CH_2_), 120.5 (C2′,6′), 122.9 (C-6), 123.3 (C-4), 128.9 (C-4′′), 129.4 (C-3′,5′), 129.5 (C-3′′), 130.0 (C-5), 132.8 (C-5′′), 133.9 (C-4′), 136.3 (C-1), 138.0 (C-3), 139.5 (C-2′′), 140.5 (C-1′), 152.6 (C=NOH), 160.7 (CONH), 165.9 (NHCO); FAB-MS *m*/*z:* 377 [M + 1 − H2O]^+^, 394 [M]^+^, 395 [M + 1]^+^.

#### 3.1.6. General Experimental Procedures of **1**–**3**

To a solution of amidoximes **1b**–**3b** (0.53 mmol) in anhydrous dichloromethane (10 mL) was added: triethylamine (1.58 mmol) and acetyl chloride (0.6 mmol) at 0 °C. The reaction mixture was stirred for 1–2 h at room temperature and ice water was added. The aqueous layer was extracted with dichloromethane (30 mL × 3). The organic phase was washed with water, saturated NaHCO_3,_ and water. The organic layer was dried with MgSO_4_, filtrated, and evaporated in reduced pressure to give the acetylated amidoximes (**1c**–**3c**), which were used next reaction without purification. To a solution of 10% Pd-C in ethanol (10 mL) was added: acetylated amidoximes (1.0 eq.) and c-HCl (1.0 eq.) and hydrogenated for 2 h at 60 psi, 45 °C. The reaction mixture was filtrated and concentrated under reduced pressure to give a crude oily compound, which was purified to yield a white or pale yellow solid by column chromatography.

*N-(3′-Amidinophenyl)-3-(thiophen-2′′-ylcarbonylamino) benzamide·HCl* (**1**). Yield: 46%; m.p.: 219–220 °C; ^1^H-NMR (400 MHz, DMSO-*d*_6_) δ: 7.23 (1H, dd, *J* = 5.0, 3.8 Hz, H-4′′), 7.55–7.50 (2H, m, H-5,4′), 7.60 (1H, t, *J* = 7.9 Hz, H-5′), 7.76 (1H, d, *J* = 8.1 Hz, H-6), 7.87 (1H, dd, *J* = 5.0, 1.0 Hz, H-5′′), 8.03 (1H, ddd, *J* = 8.2, 2.3, 1.0 Hz, H-4), 8.08 (1H, dd, *J* = 8.0, 1.2 Hz, H-6′), 8.24 (1H, dd, *J* = 3.7, 0.7 Hz, H-3′′), 8.32 (1H, t, *J* = 1.8 Hz, H-2′), 8.38 (1H, t, *J* = 1.8 Hz, H-2), 9.45 (4H, br s, hydrogens of amidine·HCl), 10.68 (1H, s, NHCO), 10.72 (1H, s, CONH); ^13^C-NMR (100 MHz, DMSO-*d*_6_) δ: 119.5 (C-2′), 120.0 (C-2), 122.7 (C-6), 123.1 (C-6′), 123.7 (C-4), 125.1 (C-4′), 128.1 (C-5), 128.7 (C-4′′), 129.0 (C-3′′), 129.3, 129.5 (C-5′), 132.1 (C-5′′), 135.0 (C-1), 139.1 (C-3), 139.7 (C-2′′), 139.8 (C-1′), 160.1 (carbon of amidine), 165.9 (CONH), 166.3 (NHCO); FAB-MS *m*/*z:* 365 [M − Cl]^+^, 388 [M − Cl + Na]^+^; HRMS (EI): Calcd for C_19_H_17_N_4_O_2_S [M + H]^+^ 365.1072, found 365.1067.

*N-(4′-Amidinophenyl)-3-(thiophen-2′′-ylcarbonylamino) benzamide·HCl* (**2**). Yield: 37%; m.p.: 237–238 °C; ^1^H-NMR (400 MHz, DMSO-*d*_6_) δ: 7.24 (1H, dd, *J* = 5.0, 3.8 Hz, H-4′′), 7.53 (1H, t, *J* = 7.9 Hz, H-5), 7.77 (1H, d, *J* = 7.9 Hz, H-6), 7.88 (1H, dd, *J* = 5.1, 1.1 Hz, H-5′′), 7.90 (2H, d, *J* = 9.0 Hz, H-3′,5′), 8.05 (3H, m, H-4, H-2′,6′), 8.21 (1H, d, *J* = 3.8 Hz, H-3′′), 8.36 (1H, t, *J* = 1.8 Hz, H-2), 9.17 (2H, br s, hydrogens of amidine·HCl), 9.36 (2H, br s, hydrogens of amidine·HCl), 10.63 (1H, s, NHCO), 10.78 (1H, s, CONH); ^13^C-NMR (100 MHz, DMSO-*d*_6_) δ: 119.7 (C-2′,6′), 120.0 (C-2), 122.1 (C-4′), 122.9 (C-6), 123.7 (C-4), 128.1 (C-5), 128.7 (C-4′′), 129.0 (C-3′′), 129.6 (C-3′,5′), 132.1 (C-5′′), 134.9 (C-1), 139.1(C-3), 139.7 (C-2′′), 144.2 (C-1′), 160.1 (carbon of amidine), 164.9 (CONH), 166.1 (NHCO); FAB-MS *m*/*z:* 365 [M − Cl]^+^, 388 [M − Cl + Na]^+^, 423 [M + Na]^+^; HRMS (EI): Calcd for C_19_H_17_N_4_O_2_S [M + H]^+^ 365.1072, found 365.1069.

*N-(4′-Amidinomethylphenyl)-3-(thiophen-2′′-ylcarbonylamino) benzamide·HCl* (**3**). Yield: 51%; m.p.: 219–220 °C; ^1^H-NMR (400 MHz, DMSO-*d*_6_) δ: 3.71 (2H, s, CH_2_), 7.23 (1H, dd, *J* = 5.0, 3.8 Hz, H-4′′), 7.44 (2H, d, *J* = 8.6 Hz, H-3′,5′), 7.50 (1H, t, *J* = 7.9 Hz, H-5), 7.71 (1H, d, *J* = 7.9 Hz, H-6), 7.79 (2H, d, *J* = 8.6 Hz, H-2′,6′), 7.87 (1H, dd, *J* = 5.0, 0.9 Hz, H-5′′), 8.02 (1H, dd, *J* = 8.1, 1.3 Hz, H-4), 8.30 (1H, t, *J* = 1.8 Hz, H-2), 8.83 (2H, br s, hydrogens of amidine·HCl), 9.28 (2H, br s, hydrogens of amidine·HCl), 10.37 (1H, s, NHCO), 10.57 (1H, s, CONH); ^13^C-NMR (100 MHz, DMSO-*d*_6_) δ: 119.9 (C-2), 120.5 (C-2′,6′), 122.7 (C-6), 123.5 (C-2), 128.1 (C-4′), 128.6 (C-4′′), 129.2 (C-3′,5′), 129.5 (C-3′′), 132.1 (C-5′′), 135.5 (C-1), 138.6 (C-3), 139.0 (C-1′), 139.8 (C-2′′), 160.1 (carbon of amidine), 165.5 (CONH), 169.3 (NHCO); FAB-MS *m*/*z:* 379 [M − Cl]^+^, 402 [M − Cl + Na]^+^; HRMS (EI) Calcd. For C_20_H_19_N_4_O_2_S [M + H]^+^ 379.1229, found 379.1216.

#### 3.1.7. General Experimental Procedures **4i**–**8i**

To a stirred solution of 3-nitrobenzoic acid (35.90 mmol) in anhydrous benzene (50 mL) was dropwise added: oxalyl chloride (46.67 mmol) and triethylamine (39.49 mmol) at room temperature. The reaction mixture was refluxed for 1 h, and benzene and unreacted oxalyl chloride were rotary evaporated off under reduced pressure and used without purification. To a solution of 4-chloroaniline, 4-bromoaniline, 4-fluoroaniline, 4-methoxyaniline, and 4-mor pholinoaniline (2.16 mmol) in anhydrous benzene (30 mL) was added: 3-nitrobenzoyl chloride (2.69 mmol) and triethylamine (2.16 mmol) at room temperature and stirred at the same temperature for 3 h. To the reaction mixture, water was added and extracted with ethylacetate (30 mL × 3), dried with anhydrous MgSO_4_, and filtrated. The filtrate was evaporated under reduced pressure to afford a crude product, which was recrystallized with ethyl acetate and *n-*hexane to give a pure white or pale yellow compound, respectively.

*N-(4′-Chlorophenyl)-3-nitrobenzamide* (**4i**). Yield: 82%; m.p.: 175–177 °C; ^1^H-NMR (400 MHz, DMSO-*d*_6_) δ: 7.45 (2H, d, *J* = 8.9 Hz, H-3′,5′), 7.82 (2H, d, *J* = 8.9 Hz, H-2′,6′), 7.85 (1H, t, *J* = 8.0 Hz, H-5), 8.40 (1H, d, *J* = 8.0 Hz, H-6), 8.45 (1H, ddd, *J* = 8.2, 2.3, 1.0 Hz, H-4), 8.78 (1H, t, *J* = 1.9 Hz, H-2), 10.71 (1H, s, CONH); ^13^C-NMR (100 MHz, DMSO-*d*_6_) δ: 122.1 (C-2′,6′), 122.4 (C-2), 126.3 (C-4), 127.8 (C-4′), 128.7 (C-3′,5′), 130.3 (C-5), 134.2 (C-6), 136.0 (C-1), 137.7 (C-1′), 147.7 (C-3), 163.4 (CONH); GC-MS (EI) *m*/*z:* 276 [M]^+^.

*N-(4′-Bromophenyl)-3-nitrobenzamide* (**5i**). Yield: 79%; m.p.: 190–192 °C; ^1^H-NMR (400 MHz, DMSO-*d*_6_) δ: 7.75 (2H, d, *J* = 8.8 Hz, H-3′,5′), 7.77 (2H, d, *J* = 8.8 Hz, H-2′,6′), 7.85 (1H, t, *J* = 8.0 Hz, H-5), 8.40 (1H, d, *J* = 7.8 Hz, H-6), 8.45 (1H, ddd, *J* = 8.2, 2.3, 1.0 Hz, H-4), 8.78 (1H, t, *J* = 1.9 Hz, H-2), 10.69 (1H, s, CONH); ^13^C-NMR (100 MHz, DMSO-*d*_6_) δ: 115.9 (C-4′), 122.4 (C-2,2′,6′), 126.3 (C-4), 130.2 (C-5), 131.5 (C-3′,5′), 134.2 (C-6), 136.0 (C-1), 138.1 (C-1′), 147.7 (C-3), 163.4 (CONH); GC-MS (EI) *m*/*z:* 320 [M − 1]^+^, 322 [M + 1]^+^.

*N-(4′-Fluorophenyl)-3-nitrobenzamide* (**6i**). Yield: 78%; m.p.: 190–191 °C; ^1^H-NMR (400 MHz, DMSO-*d*_6_) δ: 7.23 (2H, t, *J* = 8.8 Hz, H-3′,6′), 7.80 (2H, dd, *J* = 9.0, 5.1 Hz, H-2′,6′), 7.85 (1H, t, *J* = 8.0 Hz, H-5), 8.40 (1H, d, *J* = 7.8 Hz, H-6), 8.44 (1H, dd, *J* = 8.2, 2.3 Hz, H-4), 8.79 (1H, t, *J* = 2.0 Hz, H-2), 10.63 (1H, s, CONH); ^13^C-NMR (100 MHz, DMSO-*d*_6_) δ: 115.3 (*J* = 22.0 Hz, C-3′,5′), 122.4(*J* = 7.2 Hz, H-2′,6′), 122.5 (C-2), 126.2 (C-4), 130.2 (C-5), 134.1 (C-6), 135.0 (*J* = 2.6 Hz, C-1′), 136.1 (C-1), 147.7 (C-3), 158.5 (*J* = 240.0 Hz, H-4′), 163.2 (CONH); GC-MS (EI) *m*/*z:* 260 [M]^+^.

*N-(4′-Methoxyphenyl)-3-nitrobenzamide* (**7i**). Yield: 77%; m.p.: 163–164 °C; ^1^H-NMR (400 MHz, DMSO-*d*_6_) δ: 3.75 (3H, s, CH_3_), 6.96 (2H, d, *J* = 9.1 Hz, H-3′,5′), 7.68 (2H, d, *J* = 9.1 Hz, H-2′,6′), 7.83 (1H, t, *J* = 8.0 Hz, H-5), 8.39 (1H, d, *J* = 8.0 Hz, H-6), 8.43 (1H, ddd, *J* = 8.2, 2.3, 1.0 Hz, H-4), 8.78 (1H, t, *J* = 1.9 Hz, H-2), 10.47 (1H, s, CONH); ^13^C-NMR (100 MHz, DMSO-*d*_6_) δ: 55.9 (CH_3_), 114.5 (C-2′,6′), 122.9 (C-3′,5′), 123.0 (C-2), 126.7 (C-4), 130.9 (C-5), 132.4 (C-1′), 134.7 (C-6), 137.0 (C-1), 148.4 (C-3), 156.5 (C-4′), 163.5(CONH); GC-MS (EI) *m*/*z:* 272 [M]^+^.

*N-(4′′-Morpholinophenyl)-3-nitrobenzamide* (**8i**). Yield: 75%; m.p.: 204–205 °C; ^1^H-NMR (400 MHz, DMSO-*d_6_*) δ: 3.20 (4H, t, *J* = 0.8 Hz, H-3′′,5′′), 3.82 (4H, t, *J* = 0.8 Hz, H-2′′,6′′), 7.16 (2H, d, *J* = 8.4 Hz, H-3′,5′), 7.71 (2H, *J* = 9.0 Hz, H-2′,6′), 7.83 (1H, t, *J* = 8.0 Hz, H-5), 8.39–8.44 (2H, m, H-4, H-6), 8.78 (1H, t, *J* = 1.2 Hz, H-2), 10.53 (1H, s, CONH); ^13^C-NMR (100 MHz, DMSO-*d*_6_) δ: 49.9 (C-3′′,5′′), 65.6 (C-2′′,6′′), 116.7 (C-3′,5′), 121.7 (C-2′,6′), 122.3 (C-2), 126.1 (C-4), 130.2 (C-5), 134.1 (C-6), 136.3 (C-1), 147.8 (C-3), 162.9 (CONH); GC-MS (EI) *m*/*z:* 327 [M]^+^.

#### 3.1.8. General Experimental Procedures of **4ii**–**8ii**

To a solution of **4i**–**8i** (3.37 mmol) in methanol (20 mL) was added: ammonium chloride (double amounts of **4i**–**8i**) and iron powder (6.03 mmol) and refluxed for 7 h. The reaction mixture was evaporated to give an oily residue, which was added with water and extracted with dichloromethane (30 mL × 3). The combined organic phase was dried with anhydrous MgSO_4_, filtrated, and the filtrate was evaporated to yield a crude solid, which recrystallized with ethyl acetate and *n*-hexane to give a pure white or pale yellow solid.

*N-(4′-Chlorophenyl)-3-aminobenzamide* (**4ii**). Yield: 97%; m.p.: 160–161 °C; ^1^H-NMR (400 MHz, DMSO-*d*_6_) δ: 5.32 (2H, s, NH_2_), 6.75 (1H, ddd, *J* = 7.9, 2.3, 1.0 Hz, H-4), 7.04 (1H, d, *J* = 8.0Hz, H-6), 7.08 (1H, t, *J* = 2.0 Hz, H-2), 7.14 (1H, t, *J* = 7.7 Hz, H-5), 7.38 (2H, d, *J* = 8.9 Hz, 3′,5′), 7.80 (1H, d, *J* = 8.9 Hz, H-2′,6′), 10.19 (1H, s, CONH); ^13^C-NMR (100 MHz, DMSO-*d*_6_) δ: 112.9 (C-2), 114.7 (C-6), 116.9 (C-4), 121.5 (C-2′,6′), 126.9 (C-5), 128.4 (C-3′,5′), 129.8 (C-4′), 135.6 (C-1), 138.3 (C-1′), 148.8 (C-3), 166.9 (CONH); GC-MS (EI) *m*/*z:* 246 [M]^+^.

*N-(4′-Bromophenyl)-3-aminobenzamide* (**5ii**). Yield: 97%; m.p.: 176–177 °C; ^1^H-NMR (400 MHz, DMSO-*d*_6_) δ: 5.32 (2H, s, NH_2_), 6.75 (1H, ddd, *J* = 7.9, 2.2, 0.9 Hz, H-4), 7.04 (1H, d, *J* = 8.0, Hz, H-6), 7.07 (1H, t, *J* = 2.0 Hz, H-2), 7.14 (1H, t, *J* = 7.7 Hz, H-5), 7.51 (2H, d, *J* = 8.9 Hz, H-3′,5′), 7.75 (2H, d, *J* = 8.9 Hz, H-2′,6′), 10.18 (1H, s, CONH); ^13^C-NMR (100 MHz, DMSO-*d*_6_) δ: 113.0 (C-2), 114.7 (C-6), 115.0 (C-4′), 116.9 (C-4), 122.0 (C-2′,6′), 128.8 (C-5), 131.3 (C-3′,5′), 135.6 (C-1), 138.8(C-1′), 148.8 (C-3), 166.5 (CONH); GC-MS (EI) *m*/*z:* 290 [M − 1]^+^, 292 [M + 1]^+^.

*N-(4′-Fluorophenyl)-3-aminobenzamide* (**6ii**). Yield: 76%; m.p.: 143–144 °C; ^1^H-NMR (400 MHz, DMSO-*d*_6_) δ: 5.29 (2H, s, NH_2_), 6.74 (1H, ddd, *J* = 7.9, 2.3, 0.9 Hz, H-4), 7.05 (1H, d, *J* = 7.6 Hz, H-6), 7.08 (1H, t, *J* = 2.0 Hz, H-2), 7.12–7.23 (3H, m, H-5,3′,5′), 7.77 (2H, dd, *J* = 9.2, 5.1 Hz, H-2′, H-6′), 10.10 (1H, s, CONH); ^13^C-NMR (100 MHz, DMSO-*d*_6_) δ: 115.8 (C-2), 117.5 (C-6), 117.9 (*J* = 22.0 Hz, C-3′,5′), 119.6 (C-4), 124.8 (*J* = 7.8 Hz, C-2′,6′), 131.6 (C-5), 138.5 (*J* = 2.6 Hz, H-1′), 138.6 (C-1), 151.6 (C-3), 161.0 (*J* = 239.0 Hz, C-4′), 167.1 (CONH); GC-MS (EI) *m*/*z:* 230 [M]^+^.

*N-(4′-Methoxyphenyl)-3-aminobenzamide* (**7ii**). Yield: 86%; m.p.: 141–142 °C; ^1^H-NMR (400 MHz, DMSO-*d*_6_) δ: 3.73 (3H, s, CH_3_), 5.28 (2H, s, NH_2_), 6.73 (1H, dd, *J* = 7.5, 1.8 Hz, H-4), 6.90 (2H, d, *J* = 9.1 Hz, H-3′, H-5′), 7.04 (1H, d, *J* = 7.9 Hz, H-6), 7.08 (1H, t, *J* = 2.0 Hz, H-2), 7.13 (1H, t, *J* = 7.7 Hz, H-5), 7.65 (2H, d, *J* = 9.0 Hz, H-2′,6′), 9.92 (1H, s, CONH); ^13^C-NMR (100 MHz, DMSO-*d*_6_) δ: 55.1 (CH_3_), 112.9 (C-2), 113.7 (C-3′,5′), 114.6 (C-6), 116.6 (C-4), 121.8 (C-2′,6′), 128.7 (C-5), 132.5 (C-1′), 136.0 (C-1), 148.7 (C-3), 155.3 (C-4′), 165.9 (CONH); GC-MS (EI) *m*/*z:* 242 [M]^+^.

*N-(4′′-Morpholinophenyl)-3-aminobenzamide* (**8ii**). Yield: 16%; m.p.: 173–174 °C; ^1^H-NMR (400 MHz, DMSO-*d*_6_) δ: 3.06 (4H, t, *J* = 0.8 Hz, H-3′′, 5′′), 3.74 (4H, t, *J* = 0.8 Hz, H-2′′,6′′), 5.35 (2H, s, NH_2_), 6.73 (1H, d, *J* = 7.0 Hz, H-4), 6.91 (2H, d, *J* = 8.9 Hz, H-3′,5′), 7.12–7.21 (3H, m, H-2, H-5, H-6), 7.61 (2H, d, *J* = 8.9 Hz, H-2′,6′), 9.86 (1H, s, CONH); ^13^C-NMR (100 MHz, DMSO-*d*_6_) δ: 51.8 (C-3′′,5′′), 69.0 (C-2′′,6′′), 115.9 (C-2), 117.7 (C-6), 118.1 (C-2′,6′), 119.5 (C-4), 124.2 (C-3′, 5′), 131.5 (C-5), 134.5 (C-1′), 139.0 (C-1), 150.2 (C-4′), 151.3 (C-3), 168.6 (CONH); GC-MS (EI) *m*/*z:* 297 [M]^+^.

#### 3.1.9. General Experimental Procedures of **4**–**13**

To a solution of thiophene-2-carboxylic acid or 5-chlorothiophene-2-carboxylic acid (7.8 mmol) in anhydrous dichloromethane (40 mL) was added: triethylamine (8.6 mmol) and dropwise added oxalyl chloride (10.1 mmol) at room temperature. The reaction mixture was refluxed for 1 h and evaporated to remove the solvent and unreacted oxalyl chloride under reduced pressure. These acid chlorides were used for the next reaction without purification. To a solution of **4ii**–**8ii** (0.41 mmol) in anhydrous benzene (20 mL) was added: triethylamine (0.41 mmol) and prepared thiophene-2-carbonyl chloride or 5-chlorothiophene-2-carbonyl chloride (0.51 mmol) at room temperature. The reaction mixture was stirred at room temperature for 2 h and evaporated to remove the benzene. To the residue, water was added and extracted with ethyl acetate (30 mL × 3). The organic phase was dried with anhydrous MgSO_4_, filtrated, and evaporated to prepare a crude compound, which was recrystallized with ethyl acetate and *n*-hexane to give a pure white or pale yellow solid.

*N-(4′-Chlorophenyl)-3-(thiophen-2′′-ylcarbonylamino) benzamide* (**4**). Yield: 77%; m.p.: 252–253 °C; ^1^H-NMR (400 MHz, DMSO-*d*_6_) δ: 7.24 (1H, dd, *J* = 4.9, 3.8 Hz, H-4′′), 7.42 (2H, d, *J* = 8.8 Hz, H-3′, H-5′), 7.52 (1H, t, *J* = 7.9 Hz, H-5), 7.69 (1H, d, *J* = 7.8 Hz, H-6), 7.83 (2H, d, *J* = 8.9 Hz, H-2′,6′), 7.88 (1H, dd, *J* = 4.9, 0.9Hz, H-5′′), 8.01 (1H, dd, *J* = 8.1, 1.2 Hz, H-4), 8.08 (1H, dd, *J* = 3.7, 1.0 Hz, H-3′′), 8.25 (1H, s, H-2), 10.41 (1H, s, NHCO), 10.44 (1H, s, CONH); ^13^C-NMR (100 MHz, DMSO-*d*_6_) δ: 119.8 (C-2), 121.8 (C-2′,6′), 122.7 (C-6), 123.3 (C-4), 127.3 (C-4′), 128.2 (C-5), 128.5 (C-3′,5′), 128.8 (C-4′′), 129.3 (C-3′′), 132.2 (C-5′′), 135.4 (C-1), 138.1 (C-1′), 139.0 (C-3), 139.7 (C-2′′), 160.3 (CONH), 165.5 (NHCO); DIP-MS (EI) *m*/*z:* 356 [M]^+^; HRMS (EI) Calcd. For C_18_H_14_ClN_2_O_2_S 357.0465 [M + H]^+^, found 357.0457.

*N-(4′-Bromophenyl)-3-(thiophen-2′′-ylcarbonylamino) benzamide* (**5**). Yield: 65%; m.p.: 263–264 °C; ^1^H-NMR (400 MHz, DMSO-*d*_6_) δ: 7.24 (1H, dd, *J* = 7.9, 3.8 Hz, H-4′′), 7.49–7.57 (3H, m, H-5,3′,5′), 7.69 (1H, d, *J* = 6.9 Hz, H-6), 7.77 (2H, d, *J* = 8.9 Hz, H-2′,6′), 7.88 (1H, dd, *J* = 5.0, 1.0 Hz, H-5′′), 8.01 (1H, dd, *J* = 8.1, 1.2 Hz, H-4), 8.07 (1H, dd, *J* = 3.8, 1.0 Hz, H-3′′), 8.25 (1H, t, *J* = 1.8 Hz, H-2), 10.41 (1H, s, NHCO), 10.44 (1H, s, CONH); ^13^C-NMR (100 MHz, DMSO-*d*_6_) δ: 115.3 (C-4′), 119.8 (C-2), 122.2 (C-2′,6′), 122.6 (C-6), 123.3 (C-4), 128.1 (C-5), 128.8 (C-4′′), 129.3 (C-3′′), 131.4 (C-3′,5′), 132.1 (C-5′′), 135.3 (C-1), 138.5 (C-1′), 139.0 (C-3), 139.7 (C-2′′), 160.0 (CONH), 165.5 (NHCO); DIP-MS (EI) *m*/*z:* 400 [M − 1]^+^, 402 [M + 1]^+^ ; HRMS (EI) Calcd. For C_18_H_14_BrN_2_O_2_S [M + H^+^] 400.9959, found 400.9947.

*N-(4′-Fluorophenyl)-3-(thiophen-2′′-ylcarbonylamino) benzamide* (**6**). Yield: 66%; m.p.: 236–237 °C; ^1^H-NMR (400 MHz, DMSO-*d*_6_) δ: 7.20 (2H, t, *J* = 8.9 Hz, H-3′,5′), 7.24 (1H, dd, *J* = 5.0, 3.8 Hz, H-4′′), 7.52 (1H, t, *J* = 7.9 Hz, H-5), 7.69 (1H, d, *J* = 7.6 Hz, H-6), 7.80 (2H, dd, *J* = 9.2, 5.1 Hz, H-2′,6′), 7.88 (1H, dd, *J* = 5.0, 1.1Hz, H-5′′), 8.00 (1H, ddd, *J* = 8.1, 2.1, 0.8 Hz, H-4), 8.08 (1H, dd, *J* = 3.8, 1.1 Hz, H-3′′), 8.25 (1H, t, *J* = 1.9 Hz, H-2), 10.34 (1H, s, NHCO), 10.43 (1H, s, CONH); ^13^C-NMR (100 MHz, DMSO-*d*_6_) δ: 115.2 (*J* = 22.3 Hz, C-3′,5′), 119.8 (C-2), 122.1 (*J* = 7.4 Hz, C-2′,6′), 122.6 (C-6), 123.3 (C-4), 128.2 (C-5), 128.7 (C-4′′), 129.3 (C-3′′), 132.1 (C-5′′), 135.5 (*J* = 3.0 Hz, C-1′), 138.9 (C-3), 139.8 (C-2′′), 158.3 (*J* = 238.7 Hz, C-4′), 160.0 (CONH), 165.4 (NHCO); DIP-MS (EI) *m*/*z:* 340 [M]^+^ ; HRMS (EI) Calcd. For C_18_H_14_FN_2_O_2_S [M + H]^+^ 341.0760, found 341.0751.

*N-(4′-Methoxyphenyl)-3-(thiophen-2′′-ylcarbonylamino) benzamide* (**7**). Yield: 67%; m.p.: 236–237 °C; ^1^H-NMR (400 MHz, DMSO-*d*_6_) δ: 3.75 (3H, s, CH_3_), 6.93 (2H, d, *J* = 9.0 Hz, H-3′,5′), 7.24 (1H, dd, *J* = 4.6, 4.0 Hz, H-4′′), 7.50 (1H, t, *J* = 7.9 Hz, H-5), 7.64–7.72 (3H, m, H-6,2′,6′), 7.88 (1H, d, *J* = 5.0 Hz, H-5′′), 8.00 (1H, dd, *J* = 8.0, 1.6 Hz, H-4), 8.11 (1H, d, *J* = 3.7 Hz, H-3′′), 8.25 (2H, t, *J* = 1.9 Hz, H-2), 10.17 (1H, s, NHCO), 10.46 (1H, s, CONH); ^13^C-NMR (100 MHz, DMSO-*d*_6_) δ: 55.2 (CH_3_), 113.7 (C-2′,6′), 119.8 (C-2), 122.0 (C-3′,5′), 122.8 (C-6), 123.1 (C-4), 128.4 (C-5), 128.7 (C-4′′), 129.4 (C-3′′), 132.2 (C-5′′), 134.1 (C-1′), 135.7 (C-1), 138.6 (C-3), 139.8 (C-2′′), 155.6 (C-4′), 159.0 (CONH), 164.9 (NHCO); DIP-MS (EI) *m*/*z:* 352 [M]^+^ ; HRMS (EI) Calcd. For C_19_H_17_N_2_O_3_S [M + H]^+^ 353.0960, found 353.0955.

*N-(4′′-Morpholinophenyl)-3-(thiophen-2′′′-ylcarbonylamino) benzamide* (**8**). Yield: 15%; m.p.: 254–255 °C; ^1^H-NMR (400 MHz, DMSO-*d*_6_) δ: 3.07 (4H, t, *J* = 0.8 Hz, H-3′′ × 2, H-5′′ × 2), 3.74 (4H, t, *J* = 0.8 Hz, H-2′′,6′′), 6.94 (2H, d, *J* = 9.1 Hz, H-3′,5′), 7.24 (1H, dd, *J* = 4.9, 3.9 Hz, H-4′′′), 7.49 (1H, t, *J* = 7.9 Hz, H-5), 7.63 (2H, d, *J* = 9.0 Hz, H-2′,6′), 7.68 (1H, d, *J* = 7.8 Hz, H-6), 7.88 (1H, dd, *J* = 5.0, 0.9 Hz, H-5′′′), 7.98 (1H, dd, *J* = 8.1, 1.4 Hz, H-4), 8.07 (1H, dd, *J* = 3.8, 0.9 Hz, H-3′′′), 8.22 (1H, t, *J* = 1.7 Hz, H-2), 10.09 (1H, s, NHCO), 10.41 (CONH); ^13^C-NMR (100 MHz, D)MSO-*d*_6_) δ: 48.8 (C-3′′, C-5′′), 66.1 (C-2′′, C-6′′), 115.2 (C-2′,6′), 119.8 (C-2), 121.5 (C-3′,5′), 122.5 (C-6), 123.0 (C-4), 128.1 (C-5), 128.6 (C-4′′), 129.3 (C-3′′), 131.3 (C-1′), 132.1 (C-5′′), 135.8 (C-1), 138.8 (C-3), 139.8 (C-2′′), 147.5 (C-4′), 160.0 (CONH), 164.8 (NHCO); DIP-MS (EI) *m*/*z:* 407 [M]^+^; HRMS (EI) Calcd. For C_22_H_22_N_2_O_3_S [M + H]^+^ 408.1382, found 408.1372.

*N-(4′-Chlorophenyl)-3-(5-chlorothiophen-2′′-ylcarbonylamino) benzamide* (**9**). Yield: 77%; m.p.: 190–191 °C; ^1^H-NMR (400 MHz, DMSO-*d*_6_) δ: 7.29 (1H, d, *J* = 4.1 Hz, H-4′′), 7.42 (2H, *J* = 8.9 Hz, H-3′,5′), 7.53 (1H, t, *J* = 7.9 Hz, H-5), 7.71 (1H, d, *J* = 7.8 Hz, H-6), 7.82 (2H, d, *J* = 8.9 Hz, H-2′,6′), 7.96–7.99 (2H, m, H-4,3′′), 8.22 (1H, t, *J* = 1.7 Hz, H-2), 10.41 (1H, s, NHCO), 10.51 (1H, s, CONH); ^13^C-NMR (100 MHz, DMSO-*d*_6_) δ: 119.8 (C-2), 121.8 (C-2′,6′), 122.9 (C-6), 123.4 (C-4), 127.3 (C-), 128.4 (C-5), 128.5 (C-3′,5′), 128.8 (C-4′′), 129.4 (C-3′′), 134.2 (C-1), 135.4 (C-3), 138.1 (C-1′), 138.6 (C-2′′), 139.8 (C-5′′), 159.0 (CONH), 165.4 (NHCO); DIP-MS (EI) *m*/*z:* 390 [M − 1]^+^ ; HRMS (EI) Calcd. For C_18_H_13_Cl_2_N_2_O_2_S [M + H]^+^ 391.0075, found 391.0066.

*N-(4′-Bromophenyl)-3-(5-chlorothiophen-2′′-ylcarbonylamino) benzamide* (**10**). Yield: 65%; m.p.: 271–272 °C; ^1^H-NMR (400 MHz, DMSO-*d*_6_) δ: 7.29 (1H, d, *J* = 4.1 Hz, H-4′′), 7.49–7.57 (3H, m, H-5, H-3′,5′), 7.71 (1H, d, *J* = 8.9 Hz, H-6), 7.77 (2H, d, *J* = 7.9 Hz, H-2′,6′), 7.95–8.01 (2H, m, H-4,3′′), 8.21 (1H, t, *J* = 1.7 Hz, H-2), 10.41 (1H, s, NHCO), 10.51 (1H, s, CONH); ^13^C-NMR (100 MHz, DMSO-*d*_6_) δ: 116.1 (C-4′), 120.5 (C-2), 122.9 (C-2′,6′), 123.6(C-6), 124.1 (C-4), 129.1 (C-4′′), 129.5 (C-3′′), 130.0 (C-5), 132.1 (C-3′,5′), 134.9 (C-1), 136.1 (C-3), 139.2 (C-1′), 139.3 (C-2′′), 139.5 (C-5′′), 159.7 (CONH), 166.1 (NHCO); DIP-MS (EI) *m*/*z:* 434 [M − 1]^+^, 436 [M + 1]^+^; HRMS (EI) Calcd. For C_18_H_14_BrClN_2_O_2_S [M + H]^+^ 434.9570, found 434.9559.

*N-(4′-Fluorophenyl)-3-(5-chlorothiophen-2′′-ylcarbonylamino) benzamide* (**11**). Yield: 75%; m.p.: 257–258 °C; ^1^H-NMR (400 MHz, DMSO-*d*_6_) δ: 7.20 (2H, t, *J* = 8.9 Hz, H-3′,5′), 7.29 (1H, d, *J* = 4.1 Hz, H-4′′), 7.52 (1H, t, *J* = 7.9 Hz, H-5), 7.71 (1H, d, *J* = 8.0 Hz, H-6), 7.79 (2H, dd, *J* = 9.2, 5.1 Hz, H-2′,6′), 7.96–7.99 (2H, m, H-4,3′′), 8.22 (1H, t, *J* = 1.8 Hz, H-2), 10.34 (1H, s, NHCO), 10.51 (1H, s, CONH); ^13^C-NMR (100 MHz, DMSO-*d*_6_) δ: 115.9 (*J* = 21.6 Hz, C-2′,6′), 119.9 (C-2), 122.2 (*J* = 7.5 Hz, C-3′,5′), 122.9 (C-6), 123.3 (C-4), 128.4 (C-5), 128.8 (C-4′′), 129.4 (C-3′′), 134.2 (C-1), 135.4 (C-3), 135.5 (*J* = 2.2 Hz, C-1′), 138.6 (C-2′′), 138.9 (C-5′′), 158.3 (*J* = 248.8 Hz, C-4′), 159.0 (CONH), 165.3 (NHCO); DIP-MS (EI) *m*/*z:* 374 [M]^+^; HRMS (EI) Calcd. For C_18_H_14_ClFN_2_O_2_S [M + H]^+^ 375.0370, found 375.0363.

*N-(4′-Methoxyphenyl)-3-(5-chlorothiophen-2′′-ylcarbonylamino) benzamide* (**12**). Yield: 88%; m.p.: 234–235 °C; ^1^H-NMR (400 MHz, DMSO-*d*_6_) δ: 3.75 (3H, s, CH_3_), 6.93 (2H, d, *J* = 9.0 Hz, H-3′,5′), 7.29 (1H, d, *J* = 4.1 Hz, H-4′′), 7.50 (1H, t, *J* = 7.9 Hz, H-5), 7.66–7.71 (3H, m, H-6, H-2′,6′), 7.95–7.99 (2H, m, H-4,3′′), 8.21 (1H, t, *J* = 1.7 Hz, H-2), 10.16 (1H, s, NHCO), 10.52 (1H, s, CONH); ^13^C-NMR (100 MHz, DMSO-*d*_6_) δ: 55.2 (CH_3_), 113.7 (C-2′,6′), 119.8 (C-2), 121.9 (C-3′,5′), 122.8 (C-6), 123.1 (C-4), 128.4 (C-5), 128.7 (C-4′′), 129.3 (C-3′′), 132.2 (C-1′), 134.1 (C-1), 135.7 (C-3), 138.5 (C-2′′), 138.9(C-5′′), 155.5 (C-4′), 158.9 (CONH), 164.9 (NHCO); DIP-MS (EI) *m*/*z:* 386 [M]^+^ ; HRMS (EI) Calcd. For C_19_H_16_ClN_2_O_3_S [M + H]^+^ 387.0570, found 387.0565.

*N-(4′′-Morpholinophenyl)-3-(5-chlorothiophen-2′′′-ylcarbonylamino) benzamide* (**13**). Yield: 12%; m.p.: 269–270 °C; ^1^H-NMR (400 MHz, DMSO-*d*_6_) δ: 3.07 (4H, t, *J* = 0.8 Hz, H-3′′,5′′×2), 3.74 (4H, t, *J* = 0.8 Hz, H-2′′,6′′), 6.94 (2H, d, *J* = 9.1 Hz, H-3′,5′), 7.29 (1H, d, *J* = 4.1 Hz, H-4′′′), 7.50 (1H, t, *J* = 7.9 Hz, H-5), 7.63 (2H, d, *J* = 9.1 Hz, H-2′,6′), 7.70 (1H, d, *J* = 7.8 Hz, H-6), 7.94–7.96 (2H, m, H-4,3′′′), 8.19 (1H, t, *J* = 1.8 Hz, H-2), 10.09 (1H, s, NHCO), 10.49 (1H, s, CONH); ^13^C-NMR (100 MHz, DMSO-*d*_6_) δ: 49.8 (C-3′′,5′′), 55.1 (C-2′′,6′′), 115.2 (C-2′,6′), 119.8 (C-2), 121.5 (C-3′,5′), 122.7 (C-6), 123.0 (C-4), 128.3 (C-5), 128.7 (C-5), 129.3 (C-3′′), 131.2 (C-1′), 134.1 (C-1), 135.8 (C-3), 138.5 (C-2′′), 138.9 (C-5′′), 147.5 (C-4′′), 159.9 (CONH), 164.7 (NHCO); DIP-MS (EI) *m*/*z:* 441 [M]^+^ ; HRMS (EI) Calcd. For C_22_H_21_ClN_3_O_3_S [M + H]^+^ 442.0992, found 442.0983.

### 3.2. Biology

#### 3.2.1. Reagents and Instruments

Heparin was purchased from Sigma-Aldrich Co. (St. Louis, MO, USA) and tumor necrosis factor-α (TNF-α) was purchased from Abnova Co. (Taipei, Taiwan). Anti-tissue factor (TF) anti-body was purchased from Santa Cruz Biotechnology Inc. (Dallas, TX, USA) and U46619 was purchased from Calbiochem-Novabiochem Co. (San Diego, CA, USA). Factor VIIa, X, Xa, pro-thrombin, and thrombin were purchased from Haematologic Technologies (Essex Junction, VT, USA) and aPTT (APTT-XL) and PT (Thromboplastin-D) assay reagent was purchased from Fisher Diagnostics (Middletown, VA, USA). S-2222 (for Factor Xa) and S-2238 (for thrombin) were purchased from Chromogenix AB (Mölndal, Sweden). Aargatroban (direct thrombin (factor IIa) inhibitor) and rivaroxaban (direct factor Xa inhibitor) were purchased from Santa Cruz Biotechnology Inc. (Dallas, TX, USA). Microplate reader (Tecan Austria GmbH, Grödig, Austria), thrombotimer (Behnk Elektronik, Norderstedt, Germany), aggregometer (Chronlog, Havertown, PA, USA), spectrophotometer (TECAN, Männedorf, Switzerland) were used.

#### 3.2.2. Preparation of Plasma

Blood samples were taken in the morning from 10 healthy volunteers in fasting status (aged between 24 and 28 years, four males and six females) without cardiovascular disorders, allergy, and lipid or carbohydrate metabolism disorders, and untreated with drugs. All subjects gave written informed consent before participation. Healthy subjects did not use additive substances or antioxidant supplementation, and their diet was balanced (meat and vegetables). Human blood was collected into sodium citrate (0.32% final concentration) and immediately centrifuged (2000 × 15 min) in order to obtain plasma. The study protocol (KNUH 2012-01-010) was approved by the Institutional Review Board of Kyungpook National University Hospitals (Daegu, Republic of Korea).

#### 3.2.3. *In Vitro* Anticoagulant Assay

aPTT and PT were determined using a Thrombotimer (Behnk Elektronik, Norderstedt, Germany), according to the manufacturer′s instructions, as described previously [36]. In brief, citrated normal human plasma (90 μL) was mixed with 10 μL of target compounds incubated for 1 min at 37 °C, aPTT assay reagent (100 μL) was added and incubated for 1 min at 37 °C, followed by addition of 20 mM CaCl_2_ (100 μL). Clotting times were recorded. For PT assays, citrated normal human plasma (90 μL) was mixed with 10 μL of synthesized compounds stock and incubated for 1 min at 37 °C. PT assay reagent (200 μL), which had been pre-incubated for 10 min at 37 °C, was then added and clotting time was recorded. PT results are expressed in seconds and as International Normalized Ratios (INR), and aPTT results are expressed in seconds. INR = (PT sample/PT control)^ISI^, ISI = International sensitivity index.

#### 3.2.4. *In Vivo* Bleeding Time

Tail bleeding times were measured using the method described by Dejana *et al.* [37]. Briefly, ICR mice were fasted overnight before experiments. One hour after intravenous administration of synthesized compounds, tails of mice were transected at 2 mm from their tips. Bleeding time was defined as the time elapsed until bleeding stopped. When the bleeding time exceeded 15 min, bleeding time was recorded as 15 min for the analysis. All animals were treated in accordance with the guidelines for the Care and Use of Laboratory Animals issued by Kyungpook National University.

#### 3.2.5. Thrombin-Catalyzed Fibrin Polymerization

Thrombin-catalyzed polymerization was determined every 6 s for 20 min by monitoring turbidity at 360 nm using a spectrophotometer (TECAN, Männedorf, Switzerland) at ambient temperature. Control plasma and plasma incubated with synthesized compounds were diluted three times in TBS (50 mM Tris-buffered physiological saline solution pH 7.4) and clotted with thrombin (final concentration-0.5 U/mL). The maximum polymerization rate (Vmax, ΔmOD/min) of each absorbance curve was recorded. All experiments were performed three times.

#### 3.2.6. Platelet Aggregation Assay

Mouse platelets from platelet-rich plasma (PRP) were washed once with HEPES buffer (5 mM HEPES, 136 mM NaCl, 2.7 mM KCl, 0.42 mM NaH_2_PO_4_, 2 mM MgCl_2_, 5.6 mM glucose, 1% BSA (*w*/*v*), pH to 7.45). The platelet aggregation study was performed according to a previously reported method [38]. Washed plasma was incubated with the indicated concentration of compounds **1** and **2** for 3 min, and followed by stimulation with thrombin (0.1 U/mL, Sigma) and TxA_2_ analog, U46619 (2 μM) in 0.9% saline at 37 °C for 5 min. Platelet aggregations were recorded using an aggregometer (Chronolog, Havertown, PA, USA).

#### 3.2.7. Thrombin Activity Assay

Compounds **1** and **2** in 50 nM Tris-HCl buffer (pH 7.4) containing 7.5 mM EDTA were mixed with 150 mM NaCl. Following incubation at 37 °C for 2 min, factor Xa solution (150 μL; 10 U/ mL) was added, followed by incubation at 37 °C for 1 min. S-2238 as substrate (150 μL; 1.5 mM) solution was then added and absorbance at 405 nm was monitored for 120 s using a spectrophotometer (TECAN, Männedorf, Switzerland).

#### 3.2.8. Factor Xa Activity Assay

These assays were performed in the same manner as the thrombin activity assay, but using thrombin FXa (150 μL; 1 U/mL) and S-2222 (a factor Xa substrate) as substrate.

#### 3.2.9. Cell Culture

Primary HUVECs were obtained from Cambrex Bio Science (Charles City, IA, USA) and were maintained using a previously described method [39,40]. Briefly, cells were cultured until confluent at 37 °C at 5% CO_2_ in EBM-2 basal media supplemented with growth supplements (Cambrex Bio Science, Charles City, IA, USA).

#### 3.2.10. Thrombin Generation on the Surfaces of HUVECs

Measurement of thrombin generation of HUVECs was quantitated as previously described [36]. Briefly, HUVECs were pre-incubated in 300 μL containing synthesized compounds in 50 mM Tris-HCl buffer, 100 pM FVa, and 1 nM FXa for 10 min, followed by the addition of prothrombin to a final concentration of 1 μM. After 10 min, duplicate samples (10 μL each) were transferred to a 96-well plate containing 40 μL of 0.5 M EDTA in Tris-buffered saline per well in order to determinate prothrombin activation. Activated prothrombin was determined by measuring the rate of hydrolysis of S2238 at 405 nm. Standard curves were prepared using amounts of purified thrombin.

#### 3.2.11. Factor Xa Generation on the Surfaces of HUVECs

TNF-α (10 ng/mL for 6 h in serum-free medium) stimulated confluent monolayers of HUVECs (preincubated with the indicated concentrations of **1** and **2** for 10 min) in 96-well culture plates were incubated with FVIIa (10 nM) in buffer B (buffer A supplemented with 5 mg/mL bovine serum albumin (BSA) and 5 mM CaCl_2_) for 5 min at 37 °C. FXa (175 nM) was added to the cells (final reaction mixture volume, 100 μL) and incubated for 15 min. The reaction was stopped by the addition of buffer A (10 mM HEPES, pH 7.45, 150 mM NaCl, 4 mM KCl, and 11 mM glucose) containing 10 mM EDTA, and the amounts of FXa generated that were measured at 405 nm over 2 min were monitored using a microplate reader. Initial rates of color development were converted into FXa concentrations using a standard curve prepared with known dilutions of purified human FXa.

#### 3.2.12. Cell Viability Assay

MTT was used as an indicator of cell viability. Cells were grown in 96-sell plates at a density of 5 × 10^3^/well. After 24 h, cells were washed with fresh medium, followed by treatment with compounds **1** and **2**. After a 48 h incubation period, cells were washed and 100 μL of 1 mg/mL MTT was added, followed by incubation for 4 h. Finally, 150 μL DMSO was added in order to solubilize the formazan salt formed, the amount of which was determined by measuring the absorbance at 540 nm using a microplate reader (Tecan Austria GmbH, Grödig, Austria). Data were expressed as mean ± SEM of least three independent experiments.

#### 3.2.13. Statistical Analysis

Data are expressed as mean SEM (standard error of the mean) of at least three independent experiments. Statistical significance between the two groups was determined using the Student’s *t*-test. Statistical significance was accepted for *p* values < 0.05.

## 4. Conclusions

Among thirteen amidino- and non-amdinobenzamides synthesized, two amidine compounds, **1** and **2**, showed prolongation in aPTT *in vitro* and *ex vivo*, and *in vivo* bleeding time, respectively. These compounds inhibited thrombin-catalyzed fibrin polymerization and platelet aggregation induced by U46619. The activities of FXa and thrombin as well as the generation of thrombin and FXa in HUVECs were dose-dependently inhibited. This study demonstrated that the amidine group in the *meta* or *para* position of the B-ring of **1** and **2** are essential for U46619 antagonist and FXa and thrombin inhibitor. Compounds **1** and **2** seem to be promising for the development of drugs combining anticoagulant and antiplatelet activities in the same molecule. Currently, further structural optimization and the modification of this scaffold are well under way in our laboratory.

## Figures and Tables

**Table 1:** Anticoagulant activities of **1b**–**3b**, and **1**–**13** at 30 μg/mL.

Sample No.	PT (s)	aPTT (s)
DMSO ^(1)^	12.2	38.7
**1b**	-	-
**2b**	12.0	36.5
**3b**	11.9	32.9
**1**	14.9	46.9
**2**	15.4	51.9
**3**	13.5	41.4
**4**	13.8	40.2
**5**	12.8	41.7
**6**	12.0	46.0
**7**	11.8	41.0
**8**	12.5	43.0
**9**	12.8	44.0
**10**	13.0	45.1
**11**	11.4	48.2
**12**	12.7	48.9
**13**	12.2	61.3
Heparin ^(2)^	34 (10 mg/mL)	85 (0.5 mg/mL)

(-): Did not experiment. ^(1)^ DMSO was used as the negative control. ^(2)^ Heparin was used as the positive control. PT: prothrombin time, aPTT: activated partial thromboplastin time.

**Table 2:** Anticoagulant activity **1** and **2**.

*In Vitro* Anticoagulant Assay				
Sample	Dose	aPTT (s)	PT (s)	PT (INR)
Control	saline	23.6 ± 0.6	12.4 ± 0.4	1.00
**1**	5 μM	22.2 ± 0.4	12.2 ± 0.2	0.96
	10 μM	23.4 ± 0.5	12.1 ± 0.5	0.94
	20 μM	28.5 ± 0.4 *	12.2 ± 0.4	0.96
	30 μM	33.2 ± 0.7 *	13.1 ± 0.5	1.14
	40 μM	45.7 ± 0.5 *	12.8 ± 0.6	1.08
	50 μM	46.2 ± 0.6 *	12.4 ± 0.4	1.00
**2**	5 μM	23.8 ± 0.2	12.7 ± 0.5	1.06
	10 μM	22.9 ± 0.5	12.4 ± 0.4	1.00
	20 μM	31.4 ± 0.4 *	12.8 ± 0.3	1.08
	30 μM	43.5 ± 0.6 *	13.0 ± 0.6	1.12
	40 μM	50.6 ± 0.4 *	13.2 ± 0.5	1.16
	50 μM	52.1 ± 0.3 *	12.9 ± 0.6	1.10
Heparin	20 μM	53.3 ± 0.5 *	21.6 ± 0.4 *	3.79 *
	30 μM	62.5 ± 0.8 *	27.2 ± 0.6 *	6.59 *

Each value represents the means ± SEM (*n* = 5). * *p* < 0.05 as compared to control.

**Table 3:** *In vivo* bleeding time of **1** and **2**.

*In Vivo* Bleeding Time		
Sample	Dose	Tail Bleeding Time (s)
Control	saline	30.8 ± 0.8
**1**	24.1 μg/mouse	34.7 ± 1.1 *
	32.1 μg/mouse	44.1 ± 1.2 *
	40.1 μg/mouse	45.8 ± 1.0 *
**2**	24.1 μg/mouse	42.4 ± 1.0 *
	32.1 μg/mouse	51.5 ± 1.1 *
	40.1 μg/mouse	52.4 ± 1.2
Heparin	140.0 μg/mouse	51.2 ± 0.8 *
	210.0 μg/mouse	68.4 ± 1.2 *

Each value represents the means ± SEM (*n* = 5). * *p* < 0.05 as compared to control.

**Table 4:** *Ex vivo* clotting time of **1** and **2**.

*Ex Vivo* Clotting Time				
Sample	Dose	aPTT (s)	PT (s)	PT (INR)
Control	saline	32.2 ± 0.8	12.8 ± 0.4	1.00
**1**	24.1 μg/mouse	35.4 ± 1.0 *	12.9 ± 0.5	1.02
	32.1 μg/mouse	45.7 ± 0.8 *	13.1 ± 0.7	1.06
	40.1 μg/mouse	46.2 ± 0.4 *	13.4 ± 0.4	1.12
**2**	24.1 μg/mouse	41.7 ± 1.2 *	13.2 ± 0.3	1.08
	32.1 μg/mouse	49.5 ± 1.0 *	13.4 ± 0.5	1.12
	40.1 μg/mouse	51.4 ± 0.8 *	13.6 ± 0.8	1.16
Heparin	140.0 μg/mouse	63.4 ± 0.9 *	25.4 ± 0.4 *	5.18 *
	210.0 μg/mouse	75.4 ± 1.2 *	31.4 ± 0.6 *	8.62 *

Each value represents the means ± SEM (*n* = 5). * *p* < 0.05 as compared to control.
